# Health Care Fraud and Abuse: Lessons From One of the Largest Scandals of the 21st Century in the Field of Spine Surgery

**DOI:** 10.1097/AS9.0000000000000452

**Published:** 2024-06-18

**Authors:** Thomas Szewczyk, Michael S. Sinha, Jack Gerling, Justin K. Zhang, Philippe Mercier, Tobias A. Mattei

**Affiliations:** * From the Division of Neurological Surgery, Saint Louis University School of Medicine, Saint Louis, MO; † Center for Health Law Studies, Saint Louis University School of Law, Saint Louis, MO; ‡ Department of Neurosurgery, University of Utah, Salt Lake City, UT.

**Keywords:** False Claims Act, Food and Drug Administration, healthcare fraud, kyphoplasty, spine surgery, surgical innovation

## Abstract

Up to hundreds of billions of dollars are annually lost to fraud and abuse in the US health care, making it a significant burden on the system. This study investigates a specific instance of health care fraud in spine surgery, in which a medical device company ended up paying $75 million to settle violations of the False Claims Act. We review the surgical background regarding the kyphoplasty procedure, as well as its billing and reimbursement details. We also explore the official legal complaint brought by the US Department of Justice to tell the story of how one of the most significant medical innovations in spine surgery in the 21st century turned into a widespread fraudulent marketing scheme. In the sequence, we provide a detailed root cause analysis of this scandal and propose some proactive measures that can be taken to avoid such type of unfortunate events. Ultimately, this historical health care scandal constitutes a valuable lesson to surgeons, health care administrators, medical device companies, and policymakers on how misaligned incentives and subsequent unscrupulous practices can transform a medical innovation into an unfortunate tale of fraud and deceit.

## INTRODUCTION

The United States spends approximately $4.3 trillion (18% of its gross domestic product) on health care.^1^ However, 3% to 10% of this expenditure is estimated to be lost to health care fraud.^2^ Therefore, addressing the issue of health care fraud and abuse could potentially save hundreds of billions of dollars, which could be channeled back into patient care and system improvement measures. Fraud in the health care sector is not only financially burdensome but may also lead to unnecessary procedures, harm to patients, and a decline of public trust in the health care system. Fraud can manifest in various forms, including fraudulent billing for nonrendered services, upcoding to bill for more expensive procedures than those actually performed, performing medically unnecessary services, misrepresenting noncovered treatments as covered, and engaging in billing schemes that overcharge patients and insurers.^3^ All of these practices undermine the integrity of the health care system, prioritize profit over patient care, and drain valuable resources from programs intended to serve patients.

## LEGAL BACKGROUND

The US Food and Drug Administration (FDA) has regulated medical device sales since the Medical Device Amendments Act of 1976.^4^ A report analyzing the medical device industry from 1976 to 2020 noted that the passage of such an act was related to “adverse events caused by some devices… [which] have led to calls to re-examine the regulatory system for such products.”^5^ The institution of the FDA as the central regulatory agency for new medical devices represents a true landmark event in the history of public policy, which ended up deeply reshaping the US health care industry for decades to come. This truly behemoth regulatory entity, which in 2024 had a total budget of approximately $7 billion, is tasked with balancing innovation and public safety, an endeavor that requires careful oversight in ensuring the quality of new pharmaceutical, biological, medical, and cosmetic products.^6^ The key regulatory role played by the FDA regarding the pharmaceutical and medical devices market puts it under close scrutiny from industry, public officials, and citizens alike. For example, the well-known 2004 recall of Vioxx, a COX-2 selective nonsteroidal anti-inflammatory drug targeting arthritis, took place only after up to 140,000 people suffered an increased risk of serious coronary artery events secondary to the drug.^7^ Subsequent reports strongly suggested that the evidence of the drug’s risks was overlooked for years before its final recall.

Because of the high stakes associated with evaluating the safety and efficacy of new drugs and devices, the FDA regulatory approval process can be quite long. In the field of medical device sales, it has been shown that the average time from submission to approval of premarket applications for novel devices in the period from 1976 to 2020 ranged from 300 to 1000 days.^5^ The variability of inherent risks among different medical devices and categories of drugs, paired with the considerable size and heterogeneity of the medical device industry, which the FDA regulates, means that the approval process has grown more complex over time, with additional paths to market entry.

As a result of the rapid progression in the complexity of new medical and surgical equipment in recent years, as a general rule, the degree of FDA regulation of such devices tends to be less stringent than that of pharmaceuticals. As described in a collection of essays amassed from the conference marking the centenary of the FDA in 2006, the constant introduction of increasingly complex medical devices requires a continuous adaptation of the FDA regulatory strategies.^8^ Because of such an unprecedented complexity of new medical devices, historically additional regulatory oversight measures, including guardrails against fraud, have been implemented.

The False Claims Act (FCA), also called the Lincoln Law or the Informer’s Act, was first enacted in 1863 during the American Civil War in order to prevent fraudulent claims by defense contractors that provided goods and services to the Union Army.^9^ With the passage of the Medicare and Medicaid Act in 1965, the FCA became a powerful tool for the Department of Justice (DOJ) to combat health care fraud by imposing liability on individuals or entities who defraud governmental programs, including Medicare and Medicaid. Among other things, the language of the FCA, as codified at 31 U.S. Code § 3729, prohibits knowingly presenting false or fraudulent claims to the government or causing or conspiring with others to submit false claims and violations, and may result in civil penalties of up to $10,000 per claim plus fines of up to 3 times the amount of the false claim (so-called “treble damages”). It is important to highlight that the knowledge requirement under the FCA includes not only actual knowledge but also deliberate ignorance or reckless disregard of the truth (or falsity) of the submitted claim.^10^

**FIGURE 1.**
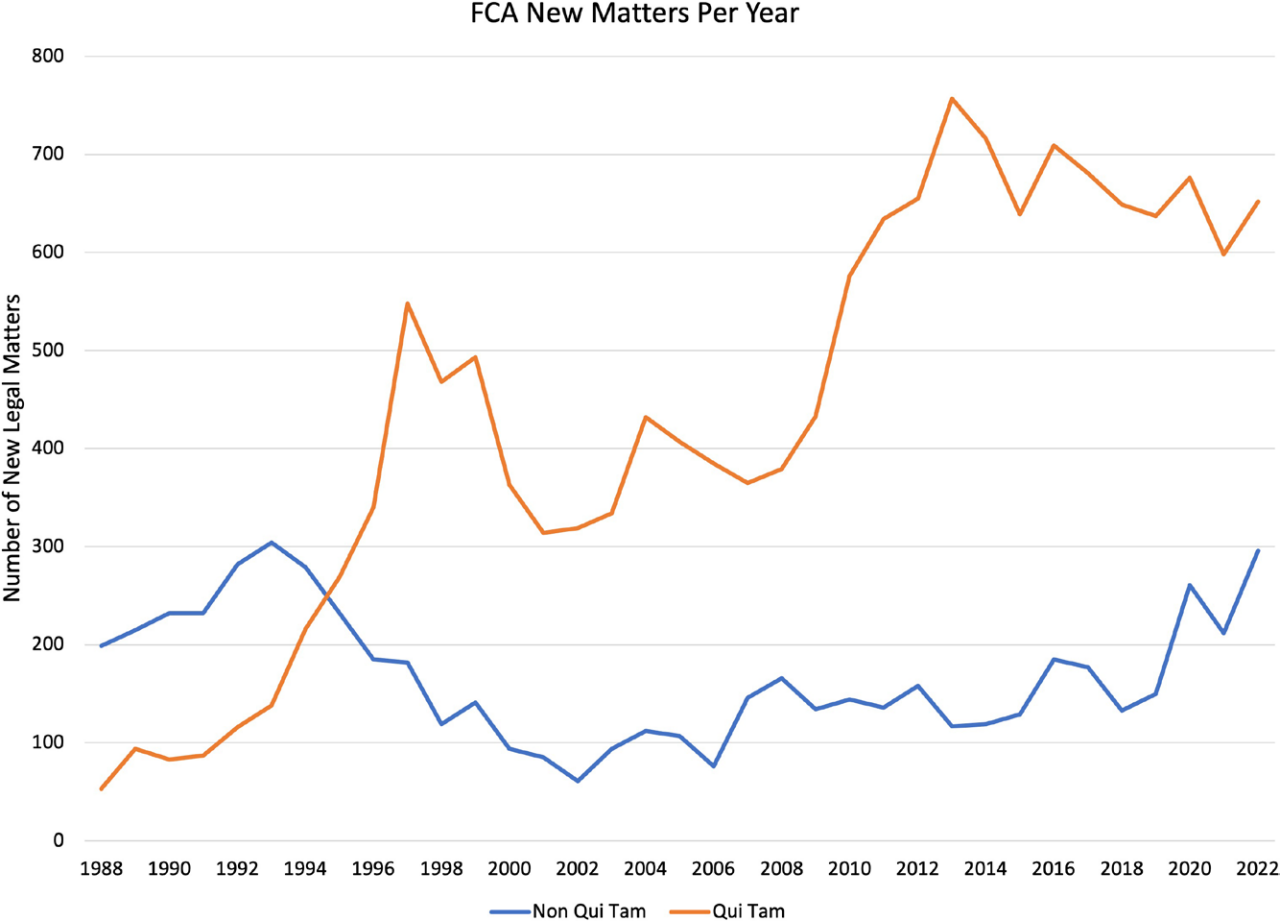
New FCA legal matters per year, including *qui tam* (orange) and non-*qui tam* (blue) matters. Data accessed from Ref. ^14^.

**FIGURE 2.**
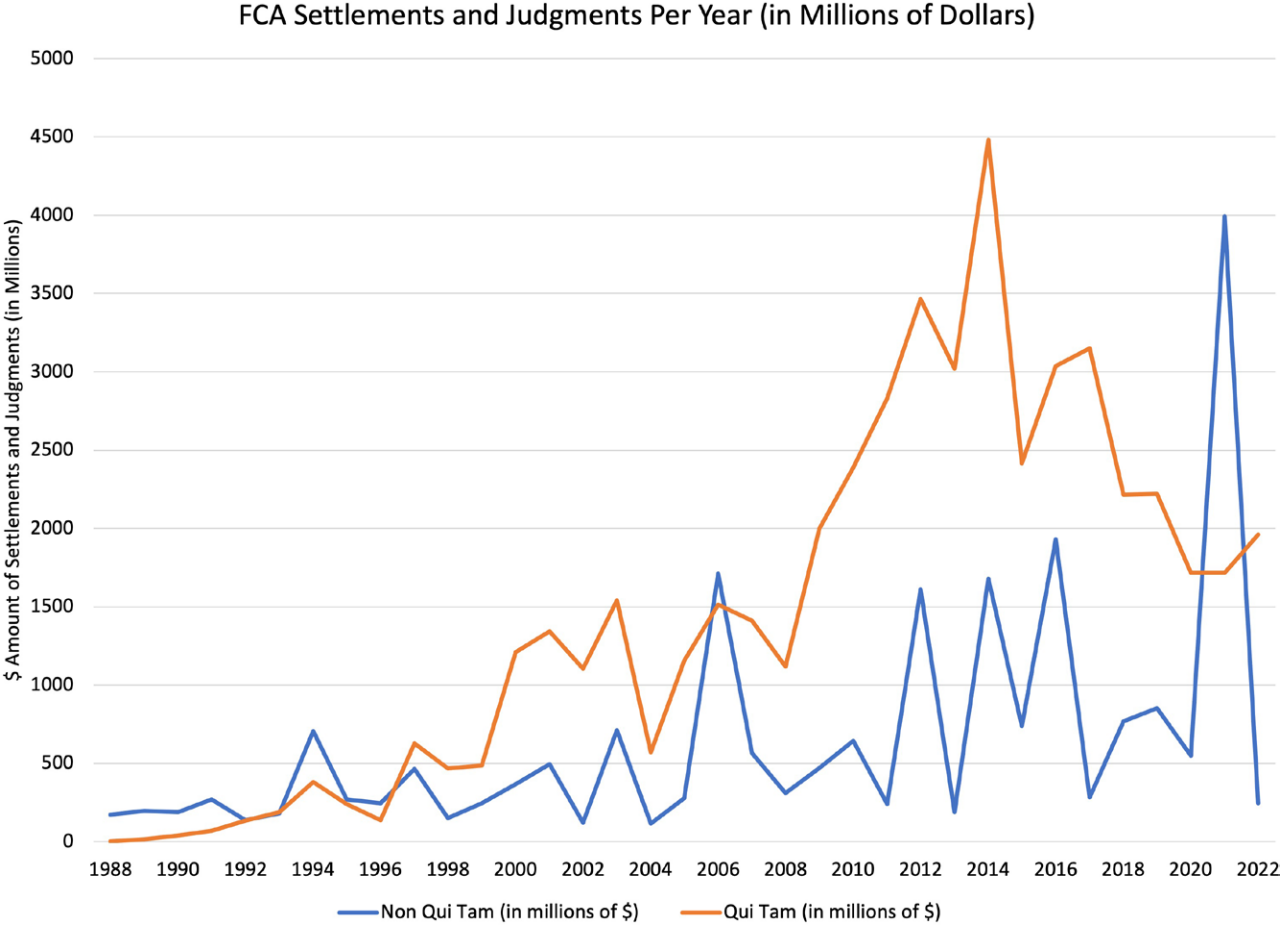
Monetary value of FCA settlements and judgments over time, including *qui tam* (orange) and non-*qui tam* (blue) matters. Data accessed from Ref. ^14^.

Through its *qui tam* provision (an abbreviation of the Latin phrase *qui tam pro domino rege quam pro se ipso in hac parte sequitur*, meaning “[he] who sues in this matter for the king as well as for himself”), the FCA allows private individuals who have knowledge about a company or individual defrauding the federal government to bring a lawsuit on behalf of the US government. The FCA was strengthened in 1986 to allow for *qui tam* relators, or whistleblowers, to receive 15% to 30% of the settlement or final judgment. Since then, the FCA has yielded significant financial recoveries, with over $72 billion (Figs. 1 and 2) in settlements.^11^ In fiscal year 2022, the DOJ reported 351 settlements and judgments under the FCA amounting to a total of $2.2 billion, with the overwhelming majority of such recovery ($1.9 billion) coming from *qui tam* lawsuits and $1.7 billion involving health care fraud.^11^ Major health care companies (pharmaceutical, medical devices, biologics, etc.) settling large amounts for violations of the FCA is not unheard of,^12^ with the largest single settlement in health care being $3 billion, which was paid in 2012 by GlaxoSmithKline LLC (GSK) for fraudulent marketing of the antidepressants Paxil and Wellbutrin.^13^

In the field of surgery, one of the most successful use of the FCA *qui tam* provision in the past decades was the so-called Kyphon scandal, which involved fraudulent marketing of the company’s kits to perform a surgical procedure known as kyphoplasty. Throughout the span of several years, increasingly aggressive marketing strategies of a company producing a medical device for a then-new procedure for the treatment of osteoporotic spinal fractures morphed into a complex and exuberant scheme designed to incite physicians to perform more of these procedures and inflate the financial gains associated with it.

In order to gain a deeper understanding of the nuances involved in FCA violations in surgical specialties and the incentive structures at fault that may render such type of fraudulent scheme feasible in the first place, we offer a detailed investigation of the Kyphon scandal, which constitutes perhaps one of the largest fraud scheme in the history of spine surgery in the 21st century, and which, up to now, has not been the subject of any in-depth analysis either in the health care law or in the spine surgery literature.

## THE KYPHOPLASTY PROCEDURE

As a complex surgical subspecialty, spine surgery has always progressed alongside technological developments in surgical instruments, implants and biologics. This inherent dependence of spine surgery upon technological innovation leads to an inevitably close connection between spine surgeons and large medical device companies that, after investing billions of dollars annually in research and development, are constantly bringing to the marketplace new devices, implants, and instruments, which may have a substantial beneficial impact on patient care and surgical outcomes. Such was the case of the kyphoplasty procedure, which has a history that is inextricably intertwined with Kyphon, the company that first developed the instrumentation required for its performance.

**FIGURE 3.**
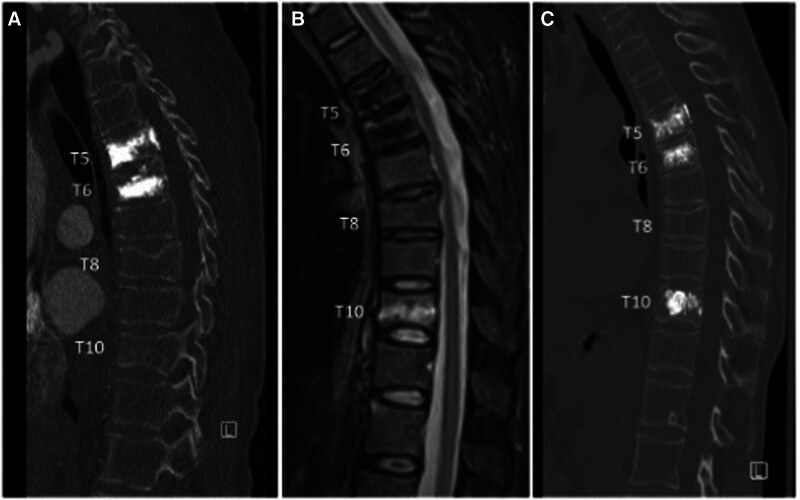
Illustrative images of a patient with an acute T10 compression fracture submitted to kyphoplasty. A, Sagittal CT scan of a patient presenting with a new onset of pain in the lower thoracic spine. Images demonstrate old T5 and T6 fractures that were previously submitted to kyphoplasties and endplate irregularities at T8 and T10; (B) STIR sagittal sequence of the MRI showing the T8 fracture to be chronic and the T10 fracture to be acute in nature. Notice the bone marrow edema at T10, which manifests as a hyperintensity in this sequence. C, Postoperative CT scan of a T10 kyphoplasty. CT indicates computed tomography; MRI, magnetic resonance imaging; STIR, short tau inversion recovery.

Osteoporosis is a condition characterized by bone fragility, ultimately leading to an increased risk of fractures, especially in the hip, spine, and wrist. Osteoporotic vertebral compression fractures (Fig. 3) more frequently occur in the thoracolumbar spine and commonly lead to vertebral body height loss, axial mechanical back pain of moderate to severe intensity, and worsening of the spinal alignment that, if occurring at multiple levels (something that is quite common in osteoporotic patients), may lead to sagittal imbalance, a condition that can substantially increase the likelihood of chronic back pain and reduce a patient’s quality of life.^15–17^ It has been estimated that the incidence of osteoporotic compression fractures in the United States is close to 700,000 cases per year.^18^ In terms of its negative impact upon a patient's health status, several studies have demonstrated that vertebral compression fractures lead to decreased mobility and increased reliance on pain medication.^15,17^

Because osteoporotic fractures are, in most cases, stable fractures, historically their treatment has involved a combination of nonsurgical measures such as bed rest, bracing, and pain medications. Introduced in France in 1984, the vertebroplasty procedure is a percutaneous technique for the treatment of osteoporotic fractures.^18^ It involves injecting bone cement (traditionally methyl methacrylate) into the vertebral body under fluoroscopic guidance. After undergoing an exothermic reaction (which by some is also believed to be responsible for its analgesic mechanism), the cement hardens inside the vertebral body, ultimately intermingling with the broken bone, restoring mechanical stability and improving the spinal pain secondary to axial load over the broken vertebra.^19,20^ In 1994 Dr. Mark Reiley, an orthopedic spine surgeon at a private practice in Boca Raton, FL, first introduced a new procedure named kyphoplasty (Fig. 3).^21^ This was a refinement of the previously described vertebroplasty. Dr. Reiley founded Kyphon, Inc. in 1994, a medical device company meant to provide necessary materials for successfully performing vertebral body cannulation and balloon inflation inside the vertebral body during a kyphoplasty procedure. For more than a decade, Kyphon was the sole company that provided instrumentation for the performance of kyphoplasty in the United States.

**FIGURE 4.**
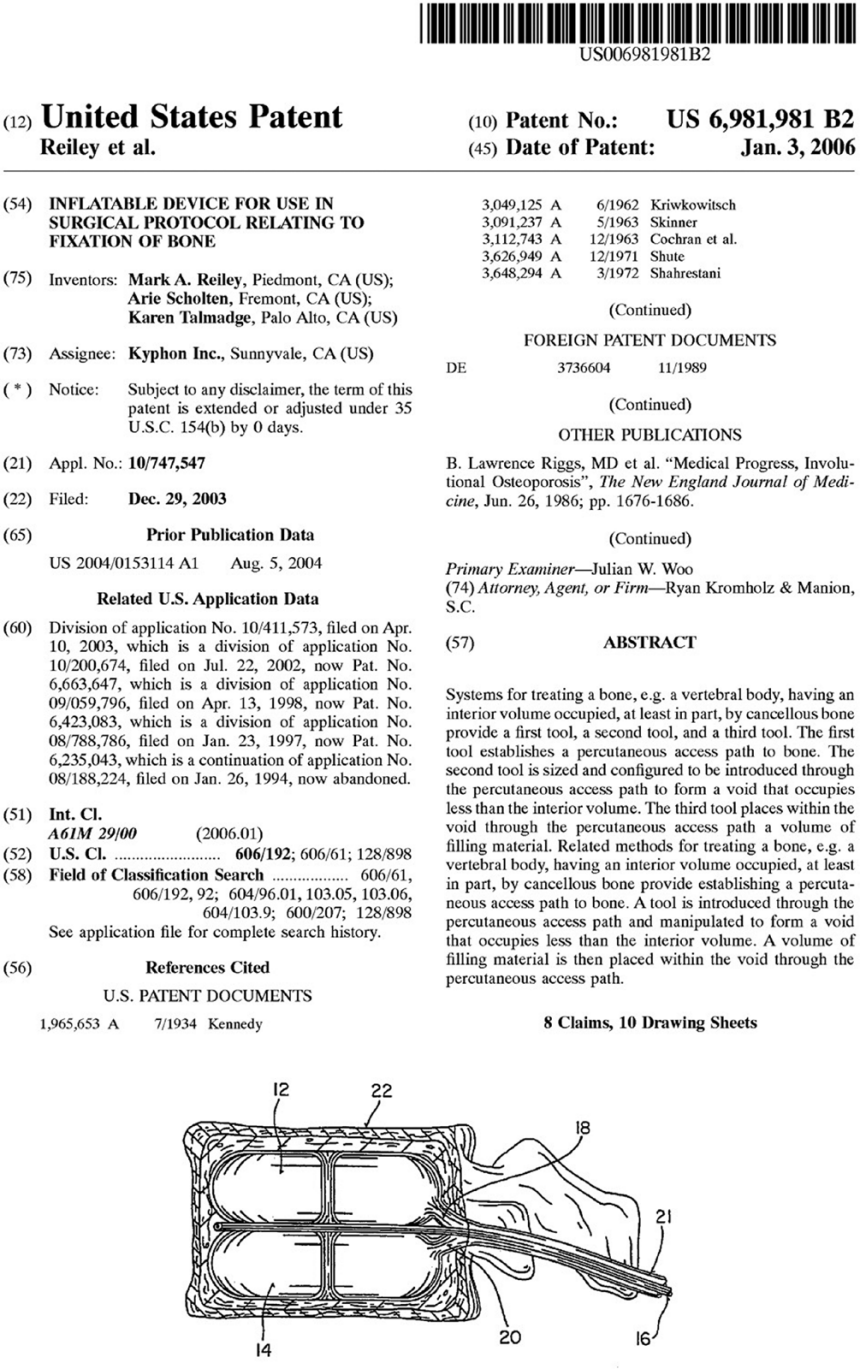
The initial patent for an inflatable balloon for reducing bone fractures (U.S. Patent No. 6,235,043) was initially granted to Kyphon in 2001 (not shown). In 2006, Kyphon obtained patent 6,981,981 (above), which was specific to balloon kyphoplasty.

The main difference between vertebroplasty and kyphoplasty is that in a kyphoplasty the surgeon employs an inflatable balloon to remodel the vertebral body and restore its height before the final cement injection (Fig. 4). There are 2 major theoretical advantages of kyphoplasty over vertebroplasty. First, it enables restoration of the vertebral body height and reduction of the segmental kyphosis associated with the initial fracture. In fact, several studies have shown kyphoplasty to be superior to vertebroplasty in terms of vertebral body height restoration.^22–24^ Second, the cavity created by the balloon allows for a low-pressure cement injection, which has been associated with lower risks of cement extravasation to critical structures such as the veins, lungs, and the spinal canal.^25^

In 2009 two clinical trials suggesting that vertebroplasty was no better than conservative treatment of osteoporotic fractures were published.^26, 27^ However, such studies later came under severe criticism, especially as the inclusion criteria did not include magnetic resonance imaging, the test that is required to differentiate between chronic fractures (for which vertebroplasty is not expected to lead to any clinical improvement) and acute/subacute fractures (the actual fractures that may benefit from such an interventional procedure). Despite such later recognized methodological flaws, the impact of these 2 studies on the cement augmentation market cannot be overstated. Suffice it to say that it led to the Medicare Services Advisory Committee pulling their financial backing of the procedure, an action that dramatically decreased the number of such procedures in the following years.

However, in 2016, high-quality data from the Vertebroplasty for Acute Painful Osteoporotic Fractures (VAPOUR) study were published.^28^ This study demonstrated the superiority of kyphoplasty over a sham procedure based on patient-reported outcome measures obtained 14 days postoperatively. Other studies have also suggested that, in patient with osteoporotic compression fractures, vertebroplasty and kyphoplasty are associated with lower long-term morbidity and mortality when compared with conservative treatment.^29–34^ Therefore, nowadays vertebral cement augmentation (in the form of vertebroplasty or kyphoplasty) is a well-established procedure for the treatment of axial mechanical back pain secondary to acute or subacute osteoporotic compression fractures.

## THE KYPHOPLASTY SCANDAL

**FIGURE 5.**
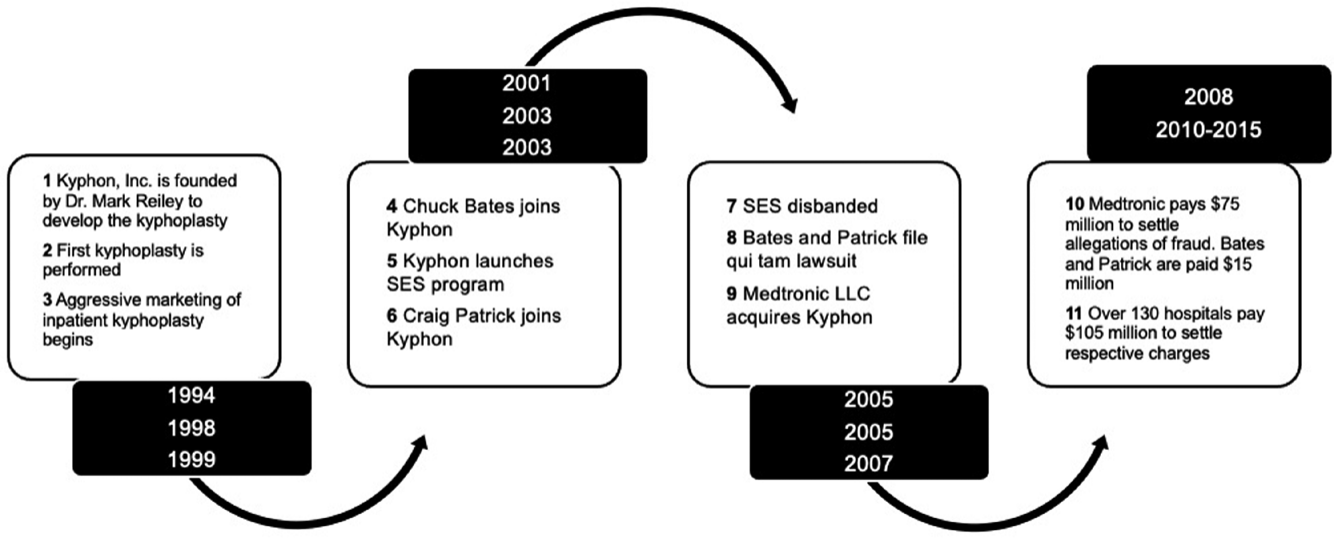
Timeline of the Kyphon scandal, from inception of the company to monetary damages following lawsuits.

Despite coming into existence as a company focused on providing the tools for a new ground-breaking interventional procedure in spine surgery, Kyphon ultimately became embroiled in a complex fraud scandal (Fig. 5), involving allegations of aggressive marketing tactics, manipulation of medical billing, promotion of unnecessary procedures, and questionable incentives provided to physicians and hospitals.^35^ Such actions were ultimately prosecuted by the DOJ under the FCA.

In order to provide a comprehensive understanding of the Kyphon scandal, in the sequence we provide a brief description of the legal claims contained in the official complaint by the DOJ against Kyphon, which contained 5 distinct allegations:

### Complaint 1: Fraudulent Marketing as an Inpatient Procedure

Kyphon’s marketing strategy, initiated in 1999, aggressively promoted kyphoplasty as an inpatient procedure requiring a hospital stay. This approach was guided by the fact that Medicare was, at the time, the largest payer for kyphoplasty, covering 85% to 90% of such procedures and its guidelines had a different reimbursement for outpatient and inpatient procedures. The result of this practice was a significant surge in the number of kyphoplasty procedures performed in the United States, with over 48,000 in 2004 and 60,000 in 2005, compared with just slightly more than 1500 in 2000. The underlying aim of Kyphon’s strategy was to maximize profit while circumventing the need to reduce the high prices of its products (at that time Kyphon’s kyphoplasty kits sold for approximately $3400). As a matter of comparison, in 2024 the Kyphon’s kyphoplasty kits cost approximately $3300. Considering the official annual inflation rates in the United States, the 2024-dollar value of the overpriced 2005 kyphoplasty kit would be approximately $5437.^36^ In other words, adjusting for inflation, the price of the Kyphon kit in 2005 was approximately 64% more expensive than its current price in 2024. The relatively low reimbursement rates available for hospitals offering kyphoplasty as an outpatient procedure were, according to internal reports from Kyphon sales representatives, the primary challenge faced by Kyphon’s in marketing its products.

Kyphon was determined not to lower the prices of its new kyphoplasty products, thereby safeguarding its substantial profit margins. However, as a percutaneous procedure, it was reasonable to infer that kyphoplasty could very likely be safely performed as an outpatient procedure in the vast majority of cases, something that stood in direct opposition to Kyphon’s aggressive promotion for its use in inpatient settings. The feasibility and safety of performing kyphoplasty in the outpatient setting were demonstrated by the history of vertebroplasty, a similar procedure that, for years, had been safely performed on an outpatient basis by interventional radiologists and spine surgeons. In fact, in terms of tissue disruption and body invasiveness, kyphoplasty was obviously closer to other outpatient percutaneous injections and bone biopsies than to open surgical procedures requiring hospital admission. However, if Kyphon was successful in convincing hospitals and physicians that this new procedure was necessarily an inpatient one, kyphoplasty could become a major cash cow for hospitals given the substantially higher reimbursement rate associated with inpatient procedures. Such a higher reimbursement would ultimately drive an increase in the annual number of kyphoplasty procedures, which would directly benefit Kyphon as, in the early days of kyphoplasty, there were no other competing companies that offered the devices necessary for performing such a procedure.

Simultaneously to its efforts in persuading health care providers and hospitals about the supposed necessity and advantage of performing kyphoplasties as inpatient procedures, Kyphon also deployed a network of paid speakers who, despite the absence of clinical data for such claims at that time, promoted kyphoplasty as a procedure with significantly lower complication rates than vertebroplasty. Physicians billing kyphoplasty as an inpatient procedure were chosen as Kyphon “faculty” members and received speaker fees for their services, aiding in a concerted effort to incentivize health care professionals to support Kyphon’s views. In contrast to such a categorical view adopted by Kyphon regarding the absolute need for kyphoplasty to be performed in the inpatient setting, prevailing Medicare guidelines at the time recommended physicians to judge the necessity of inpatient admission based on a comprehensive assessment of each patient’s comorbidities, the need for additional diagnostic studies, the magnitude of the surgical procedure in question, and the likelihood of any potentially serious complications that could be avoided by continuous monitoring in an inpatient setting.

The DOJ maintained that, in most cases, inpatient status after kyphoplasty was medically unnecessary, and Kyphon was aware of this fact. Despite its awareness of such facts, Kyphon adopted a fraudulent marketing campaign to persuade hospitals and physicians that kyphoplasty necessitated inpatient admission. Such a marketing strategy was ultimately centered on the manipulation of the Diagnosis-Related Group (DRG) reimbursement scheme to ensure higher revenue for target hospitals through unnecessary inpatient stays, thereby disregarding the genuine medical necessity of such hospital admissions.

Starting in 1999, Kyphon’s sales and marketing departments purposefully trained their representatives to aggressively market kyphoplasty as an inpatient procedure requiring a one-night hospital stay. Kyphon managers, including national sales representatives, like Chuck Bates (who later became a *qui tam* realtor), emphasized the profitability of inducing physicians to perform kyphoplasty as an inpatient procedure, even for patients with a limited number of comorbidities. For cases requiring overnight observation, outpatient admission for up to 23 hours was available, with bills submitted under Medicare Part B. However, Kyphon’s inpatient strategy led to patients’ stays and hospitals being reimbursed under DRG codes, which reimbursed substantially more than outpatient or 23-hour observation procedures.

DRGs are set by Medicare based on the resource needs of the average patient with a particular set of diseases or disorders and can substantially increase the total hospital reimbursement, especially when considering the elderly population that received the vast majority of kyphoplasty procedures.^37^ Kyphon sales representatives worked to ensure hospitals admitted patients under DRGs 233 and 234, which paid hospitals $6000 to $10,000 per procedure. Typical patient stays under these DRGs averaged 12 to 14 days (DRG 233) and 6 to 8 days (DRG 234) in stark contrast to billing kyphoplasty as an outpatient procedure, which netted a hospital less than $50 (though that reimbursement rose to $2000 in 2005). Thus, hospitals earned thousands more per patient by simply and unnecessarily admitting kyphoplasty patients.

Sales training focused heavily on reimbursement, with techniques to counter hospital objections often discussed in regional and area teleconferences. In some cases, when hospital utilization review and case management personnel objected to inpatient admissions, sales representatives argued that inpatient status was medically necessary for patient safety and that outpatient treatment was substandard. Published articles of dubious scientific quality authored by paid consultants for the company were distributed to justify inpatient admission.

Ultimately, a significant number of hospitals appear to have embraced Kyphon’s proposition of increased revenue through inpatient procedures, fostering a symbiotic relationship that financially benefited both parties. Kyphon created an “Economic Support Model” to demonstrate to hospitals that they should not lose money on inpatient kyphoplasty procedures. The model assumed that all kyphoplasty procedures would be performed inpatient and did not even consider the possibility of outpatient reimbursement. In several documented instances, sales representatives were present in the operating room during kyphoplasty procedures and took steps to ensure inpatient admission by influencing physicians, residents, and nurse practitioners responsible for postoperative orders. It was alleged that sales representatives often misled physicians by falsely claiming that outpatient reimbursement was unavailable from Medicare for such a procedure.

### Complaint 2: Fraudulent Marketing Reimbursement for Unnecessary Bone Biopsies

Around 2001, Kyphon sales representatives began marketing to physicians and hospitals the practice of routinely performing bone biopsies during kyphoplasty procedures, even though, in the vast majority of cases, such procedures were being performed for benign osteoporotic compression fractures with no underlying suspicion of malignancy. Obtaining such biopsies allowed billing under DRG 216, which could lead hospitals to earn up to $6000 more per inpatient procedure depending on the geography, or up to $9500 more if the addition of a bone biopsy resulted in inpatient admission. Sales representatives encouraged doctors to perform biopsies on every patient, irrespective of medical necessity. In February 2003, Kyphon introduced the “Bone Biopsy Device,” intended for taking biopsy samples of bone during the procedure and which sold for $110. The purpose of this new biopsy device was twofold: to provide a better means of bone biopsy and to foster hospital reimbursement under DRG 216.

Kyphon’s strategy involved promoting the bone biopsy procedure through “scare” tactics, suggesting that physicians who did not routinely perform biopsies could face malpractice suits. Sales representatives were trained to emphasize, based on nonexisting data, that up to 4% of biopsies could diagnose a previously undiagnosed cancer.

Although Medicare only reimburses for a biopsy when it is medically necessary, Kyphon’s strategy was to persuade doctors to perform biopsies on every patient aiming to get the Bone Biopsy Device listed as a preferred equipment choice in the operating room for surgeons. The marketing of the bone biopsy procedure was widely successful and increased enthusiasm about better reimbursement for kyphoplasty on the part of many hospitals.

### Complaint 3: Encouraging Upcoding

Between 1999 and 2005, Medicare carriers had varying policies regarding kyphoplasty coverage. Some carriers covered the procedure and advised providers to bill it under an unlisted code, while others initially refused to cover it or reviewed cases individually. In regions with available coverage, many surgeons were hesitant to use the Current Procedural Terminology (CPT) Code 22899 (meant for “other spine procedures”), as unlisted codes triggered automatic review by Medicare carriers. Kyphon’s sales representatives were trained to encourage doctors and their office staff to bypass coverage determinations and explore “alternative methods” of billing to increase reimbursement.

Sales managers instructed sales representatives to persuade physicians that billing the procedure as an “open reduction of a spinal fracture” was the most effective method for reimbursement, although anyone familiar with such a percutaneous procedure can attest that no reasonable surgeon would classify it as an “open procedure.” Training presentations described the “open treatment and/or reduction” codes as “potentially related” CPT codes. Sales training materials guided representatives to advise physicians that, although kyphoplasty could be billed under an unlisted code, greater profit could be earned (allegedly through lawful means) by using CPT codes 22327, 22325, and 22328 for open reduction of thoracic or lumbar fractures. They were also trained to misrepresent to doctors that it was acceptable to bill kyphoplasty under these codes because they were “reducing” the fracture by inflating the balloon, despite the fact that such an interpretation completely ignores the “open” aspect required by these codes.

Billing kyphoplasty under CPT codes for open reduction of spinal fractures allowed physicians to obtain coverage of claims even from carriers with “no coverage” policies while avoiding claim reviews triggered by billing under the unlisted code or having to petition for case-by-case coverage. Information about the “propriety” of billing kyphoplasty procedures under “open reduction codes” was disseminated directly and indirectly by Kyphon. Sales representatives guided doctors on how to bill for the procedure and directed them to contact doctors already using open reduction codes.

### Complaint 4: Marketing Surgeon Practices to Obtain Referrals

According to the DOJ complaint, physicians were routinely given tangible and in-kind services to encourage them to perform kyphoplasty procedures. Sales representatives made promises to market the practices of certain key physicians, as well as the kyphoplasty procedure itself, to referring physicians. They also assured that the physician’s name would be mentioned in talks with senior groups and in various forms of advertising, such as newspapers.

In early 2003, Kyphon initiated the Spine Education Specialist (SES) program to extend the efforts of its sales representatives by providing direct marketing support to key physicians. The SES program was designed to cultivate relationships with referral sources and identify eligible patients for kyphoplasty referrals to trained surgeons. The program identified key surgeons and assigned SES representatives to work with them. These representatives would then identify physicians likely to refer patients to the target surgeon. The SES representatives would build relationships with primary care physicians and their staff, assist in reviewing patient charts, and help to identify eligible patients to refer to the surgeons performing kyphoplasty. Kyphon selectively placed SES representatives on target locations based on various criteria, such as states with high surgeon reimbursement, territories with a significant number of active surgeons, top prescribing primary care physicians, tenured spine consultants with excellent physician relationships, and areas with high growth potential.

The SES program significantly enhanced revenue growth for both Kyphon and Kyphon-trained doctors. SES representatives were rewarded for their referral work, effectively acting as a patient “mining” force to boost kyphoplasty referrals. Their compensation packages included quotas and commissions based on the number of new kyphoplasty procedures performed as a result of such referrals. Interventional radiologists who went through the SES program were said to have noted its positive impact, likening it to having “an extra employee.” Kyphon facilitated this process by providing the SES representatives with a “high subscriber” spreadsheet listing 19,000 prescribers of osteoporosis medications.

After internal concerns were raised by *qui tam* realtor Craig Patrick regarding Kyphon’s payment to generate referrals, the SES program was disbanded. However, Kyphon sales representatives continued their referral and marketing support activities. Finally, Kyphon also provided other inducements to high-performing physicians, such as paying them to be part of the Kyphon physician faculty and to speak to other doctors at Kyphon events, running ads promoting their practices in newspapers, and offering various gifts, including expensive bottles of wine and dinners for physicians and their spouses.

### Complaint 5: Hospital Inducements

Kyphon engaged in a practice of providing free products to hospitals in order to induce them to select kyphoplasty rather than vertebroplasty as the preferred procedure at their facilities.

Sales representatives also offered free product samples to hospitals as an incentive to purchase Kyphon products. They gave away sample “kits” to hospitals, with no specific limit on the number of kits they could provide, as long as they tracked their samples. Sales representatives even requested “No Charge Purchase Orders” from hospitals to demonstrate the amount of free products received and the cost savings realized by using these free products. In one instance, a Kyphon Spine Consultant disclosed that Kyphon provided free kits to a medical center when medically necessary to perform kyphoplasty on more than one vertebral level, a practice that generated a pernicious incentive to perform multilevel kyphoplasty procedures even when the second fracture was minor and, by itself, would not require intervention. This practice was endorsed by Kyphon management at the time.

Kyphon’s offered free kits to hospitals in order to reduce their costs and increase reimbursement for each kyphoplasty procedure performed; yet under the Anti-Kickback Statute, claims from hospitals that have received kickbacks are considered ineligible for reimbursement by Medicare.

## WHISTLEBLOWERS FILE A QUI TAM ACTION

**FIGURE 6.**
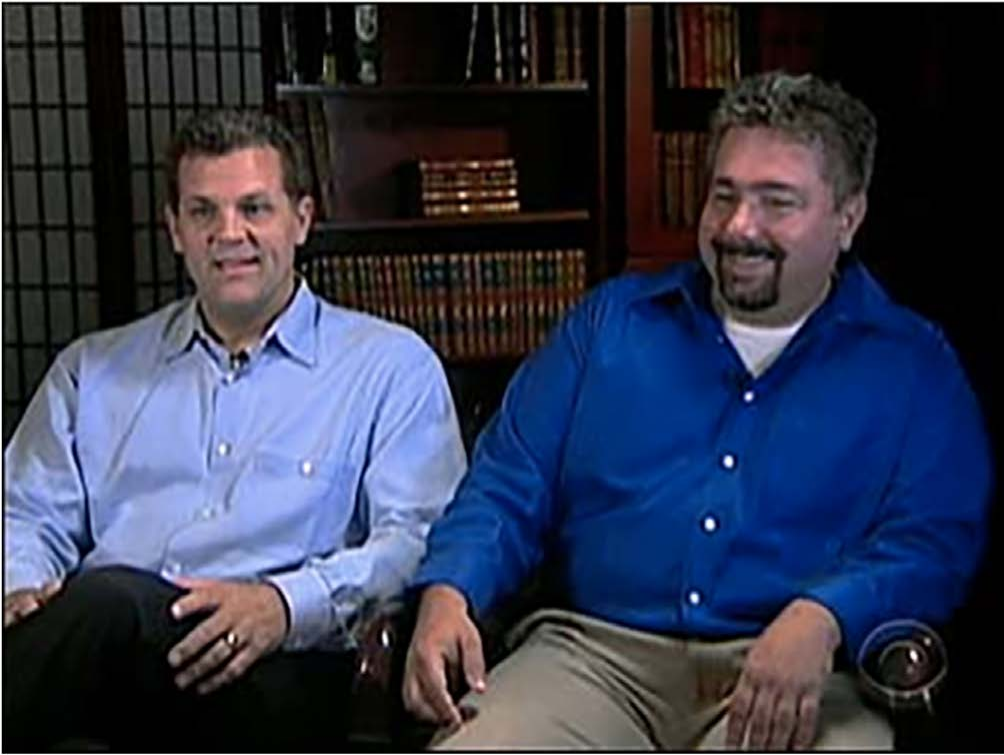
Chuck Bates (left) and Craig Patrick (right) are interviewed on CBS regarding their role as whistleblowers following Kyphon’s settlement with the DOJ. Taken from Ref. ^38^.

Two whistleblowers, Chuck Bates and Craig Patrick (Fig. 6), played a pivotal role in the DOJ case against Kyphon. Bates started working as a sales representative for Kyphon in 2001 and was responsible for establishing and developing key relationships with physicians and hospitals, providing instructions on how to bill for the procedure as inpatient and as an “open reduction.” Bates attended training meetings, the sole purpose of which was to promote kyphoplasty as an inpatient procedure. After growing business in his Southern territory from $16,000 per month to $200,000 per month, Bates was promoted to Kyphon Regional Sales Manager in 2002.^35^

Craig Patrick worked as a reimbursement manager focused on improving insurance coverage and reimbursement of kyphoplasty procedures. Among the several alarming practices, the promise of free kyphoplasty kits in exchange for certain favors is said to have impressed him. For example, a hospital in Nashville, TN, initially decided against allowing physicians to perform kyphoplasty as an outpatient procedure due to its poor reimbursement. Kyphon responded by promising free kyphoplasty kits for all outpatient procedures under the condition that they begin performing some procedures as inpatient as well. In another example, while talking about CPT billing at a company presentation to physicians, Patrick was interrupted and “corrected” by other sales representatives, who emphasized that kyphoplasty should be billed under the aforementioned “open reduction” CPT codes.

Patrick and other reimbursement managers sent out multiple memos within the company regarding concerns about kickbacks and other dubious marketing practices. In 2005, Bates and Patrick filed a *qui tam* lawsuit against Kyphon, which prompted a comprehensive investigation by the DOJ.

## THE AFTERMATH

As a result of Kyphon’s marketing scheme, between 80% and 90% of the 150,000 kyphoplasty procedures performed between 1999 and 2005 were done on an inpatient basis.^35^ During those 6 years over 4500 physicians in the United States were treating spinal fractures with Kyphon’s products.^39^ Despite a previous patent infringement battle between the 2 companies,^40^ in 2007, Medtronic Spine LLC acquired Kyphon, but also knowingly inherited the legal ramifications of their predecessor’s criminal actions, including 3 counts of fraud under the FCA.^32,33^ The following year, Medtronic agreed to pay $75 million to the Medicare Trust Fund to settle allegations of Kyphon’s fraudulent practices.^41^ Today, Medtronic is valued at over $90 billion.^42^ The whistleblowers, Bates and Patrick, were awarded $15 million under the FCA, illustrating the role of *qui tam* claims in stimulating and properly rewarding successful claims of health care fraud.^41^ More than 130 hospitals were also implicated in the scheme and collectively paid the government $105 million to settle charges related to the Kyphon scandal.^43^ One physician, an orthopedic surgeon from New York, settled and paid $388,000 for his alleged violations of the FCA.^44^

## LESSONS LEARNED

Unfortunately, fraudulent billing and dubious coding practices are not uncommon occurrences in modern medical practice. Increased revenues for upcoding of procedures can be alluring and despite mandatory training in compliance, best practices, and health care policies, physicians and hospitals are often unaware of (or perhaps decide to simply ignore) the repercussions of engaging in such reproachable practices. Though the FCA was originally enacted in response to defense contractor frauds in the mid-19th century, the passage of Medicare and Medicaid Act in 1965 meant that a significant portion of future health care spending would come from the federal government. Upcoding for procedures would therefore, in a substantial number of cases, constitute an act of defrauding the federal government through excess payments for health care services. Federal expenditures for health insurance continued to grow after the passage of the Affordable Care Act in 2010.

By and large, physicians do not purposefully set out to defraud the government, but they are notoriously prone to influence by industry. For spine surgeons, one of the main ways this influence manifests is in the form of medical device representatives, who are often in the operating room during procedures and with whom close relationships are often formed. In the Kyphon example, such close relationships clearly influenced physician decision-making and obscured sound practices, ultimately generating excess billing to federal payers. As a general rule, surgeons should strive to engage in arms-length interactions with industry representatives, particularly when it comes to determinations that may be financially lucrative albeit legally questionable. Because the FCA can penalize actual knowledge, deliberate ignorance, or reckless disregard, a surgeon who takes actions in reliance on input from industry representatives may end up liable for such actions.

This is even more important when considering new surgical procedures for which the underlying billing framework may not be clear in the early years after such technological innovations appear in the market. Perhaps there is a period after the inception of an innovative medical procedure but before a reasonably adjusted billing protocol is instituted, which enhances the incentive for fraudulent billing. All parties involved should be aware of this period and work to shorten it, perhaps via establishing a conduit from physicians to regulators to give input on how DRG codes (updated annually by Medicare) affect patient care.

Ultimately none of the major fraud concerns in this case were related to missteps in the FDA’s regulatory or approval process of the kyphoplasty technology, but were rather related to the unscrupulous business model adopted by the parent medical company. The scenario involved a completely new surgical treatment (kyphoplasty), for a widely prevalent condition (osteoporotic fractures) in a marketplace in which Kyphon was the only commercial entity providing such a product. Kyphon then took advantage of a regulatory environment that lacked both the necessary reimbursement standards as well as close scrutiny by disinterested parties representing key stakeholders in this process (especially patients, insurance companies, and government payers). This combination of factors was the perfect recipe for widespread health care fraud.

The Kyphon scandal also points to the important role of whistleblowers in combating waste and fraud in the health care system. The 1986 update to the FCA created financial incentives for whistleblowers and serves as a clear example of incentives that are positively aligned, where both industry members and regulators are rewarded for standing against fraud.

This scandal also highlights the dangers of health care fraud associated with new surgical procedures that may be indicated for a broad swath of the population, especially when there are no other competitors in the market. In such situations, taking into account the immense financial rewards that lurk on the horizon, it is conceivable that a single company may decide to pursue questionable practices in the name of profit. When there are no competitors to counterbalance such actions and when the very billing framework may still be poorly adapted to the new procedure, such an environment may yield some gray zones that may be intentionally and unethically manipulated by profit-seeking individuals occupying positions of power in the health care industry.

Surgical innovations have been critically important to the advancement of medicine and surgery, but fraud and abuse laws play a crucial role as a countermeasure to the pervasive marketing of products and procedures by pharmaceutical and medical device companies, which often occurs through representatives who have more training in marketing than science. The Kyphon scandal demonstrates that surgeons are not immune from industry pressure and may be held liable for overbilling the federal government, even if the main driver of the conduct was an industry representative. Finally, the Kyphon scandal illustrates how surgeons should be keenly aware of the importance of comprehensive training not only in the technical aspects of a new surgical procedure but also in the nuances of proper billing in order to mitigate the risk of fraudulent practices. As the “captain of the ship,” surgeons must discern the appropriate from the excessive, particularly when the consequences can be so dire.

## CONCLUSIONS

The Kyphon scandal serves as a poignant reminder of the damage that health care fraud can inflict on the system and its beneficiaries. By understanding the historical events and alleged criminal actions in this case, surgeons can gain important insights on how to better safeguard the health care system from such nefarious practices in the future. What started as an important medical innovation in the field of spine surgery evolved into a widespread national fraudulent marketing scheme from which both hospitals and Kyphon profited handsomely. By perpetuating the simple lie that this operation required an inpatient stay (and later coupling other unethical practices to this peddling scheme), hospitals and Kyphon set up a scheme to win (to the tune of tens of millions of dollars) while taxpayers lost. This unfortunate episode, which likely represents one of the largest fraud scandals in the field of spine surgery in the 21st century, should serve as a cautionary tale to all of us. Institutions, regulatory agencies, and physicians alike should take proper measures to prevent such loopholes in the health care system as well as to discourage practitioners and hospitals from engaging in such lucrative schemes.

## ACKNOWLEDGMENTS

T.S., J.G., J.K.Z., and T.A.M.: Participated in the writing of the paper and in research design. M.S.S.: Participated in the writing of the paper. P.M.: Participated in the writing of the paper.
